# Using an Instagram campaign to influence knowledge, subjective norms, perceived behavioral control, and behavioral intentions for sustainable behaviors

**DOI:** 10.3389/fpsyg.2024.1377211

**Published:** 2024-07-31

**Authors:** Alexander Varni, Chan L. Thai, Sandra Jamaleddine

**Affiliations:** Department of Communication, Santa Clara University, Santa Clara, CA, United States

**Keywords:** climate change, theory of planned behavior, behavior change intervention, university students, Instagram

## Abstract

**Introduction:**

Climate change poses one of the most pervasive threats to the planet today. Intervention is required to promote pro-environmental behaviors among individuals to curb its effects. Borrowing several constructs from the Theory of Planned Behavior, we designed and evaluated a campaign, delivered primarily through Instagram, to shift sustainability-related cognitions and behaviors among university students.

**Methods:**

An online survey was distributed to undergraduate students at a Northern California university and collected responses from 1,552 participants.

**Results:**

Comparing students who self-reported exposure to the campaign with those who were not exposed, students who observed the campaign materials had more knowledge about (*p* < .001), greater perceived social norms about (*p* < .001), and greater intentions to perform sustainable behaviors (*p* < .001). There were also increases in sustainable behaviors during the campaign, compared to the previous academic term.

**Discussion:**

Implementing campaigns inspired by behavior change theories may be one viable strategy to increase individual sustainable behaviors for climate change mitigation.

## Introduction

One of the greatest crises facing our planet today is the threat posed by global climate change. The harmful effects of climate change range from sea level rise and biodiversity loss to increases in the frequency of extreme weather events and the spread of infectious diseases (Intergovernmental Panel on Climate Change, 2019, 2020, 2021; World Health Organization, 2020). Climate change also presents numerous threats for human communities, which range from immediate threats, like injury, death, and property damage associated with extreme weather events, to more prolonged threats: like decreases in agricultural productivity and food shortages associated with prolonged droughts (Intergovernmental Panel on Climate Change, 2019, 2020, 2021).

Mitigation measures have not kept up with these rapid changes. While institutional policy solutions play an important role in improving global sustainability practices, the actions of individuals can also greatly contribute to sustainability efforts (Intergovernmental Panel on Climate Change, 2014, 2019). Thus, motivating individual behavioral change en masse will be an important aspect of meeting sustainability goals (Intergovernmental Panel on Climate Change, 2014). Taking into account readiness levels for behavior change and shifting the cognitive processes underlying behavior change is one possible route toward more sustainable behaviors (Wakefield et al., 2010). College students may have more positive attitudes surrounding sustainable behaviors (Carducci et al., 2021), and may hold heightened levels of awareness about climate change (Mazo and San Juan, 2019). Further, recent research has suggested that college students may be an important audience for pro-environmental behavior interventions, and that educational institutions could play a role in motivating sustainable behaviors (Yusliza et al., 2020; Kousar et al., 2022; Song et al., 2022). A greater understanding of what types of interventions are effective in promoting pro-environmental behaviors among individuals is needed. Accordingly, the purpose of this study is to evaluate a virtual campaign, which borrowed several constructs from the Theory of Planned Behavior, designed to encourage sustainable behaviors among college students.

## Literature review

### Individual behaviors for sustainability

Individual actions can be taken to improve sustainability, such as reducing food waste, eating plant-rich diets, taking public transit, carpooling, and recycling (Intergovernmental Panel on Climate Change, 2019, 2020; Landholm et al., 2019; Murray and DiGiorgio, 2021). These strategies are helpful and important, but they are not being implemented at great enough scales (Cotton et al., 2016; Intergovernmental Panel on Climate Change, 2019, 2020; Murray and DiGiorgio, 2021). Thus, mass communication interventions that could potentially shift a large group of people to engage in sustainability behaviors are necessary to create the scale of change needed to address this crisis. Mass media campaigns have been used to successfully alter the health behaviors of target populations, such as smoking (Bala et al., 2008; National Cancer Institute, 2008), cancer screenings (Baron et al., 2008), and heart disease prevention (Fortmann et al., 1986), among others (Wakefield et al., 2010). Their use for encouraging sustainability-related behaviors is a burgeoning area of research. Reaching receptive audiences who may be more ready to change can be a key factor to success in behavior change campaigns (Prochaska and Velicer, 1997).

### College students and sustainability

Undergraduate university students are an important target audience that may be more receptive to changing their behaviors in relation to climate change (Sinatra et al., 2012; Li, 2014; Kousar et al., 2022; Li et al., 2022; Song et al., 2022). Several studies suggest that undergraduate students who are exposed to information about the climate crisis may: (1) hold increased awareness of the issues associated with climate change, and (2) be open to learning more about climate change (Sinatra et al., 2012; Aksit et al., 2017). Furthermore, some research has shown a connection between increased understanding of climate change and increased sustainability behaviors among university students (Sinatra et al., 2012; Mazo and San Juan, 2019; Kousar et al., 2022), and has identified the potential of educational programs and interventions to motivate sustainable behaviors among students (de Vreede et al., 2014; Li, 2014; Yusliza et al., 2020; Li et al., 2022). Sinatra et al. (2012) found that when exposed to IPCC data, students were more receptive to learning about the topic and more willing to take action to reduce their individual carbon footprints. Kousar et al. (2022) echoed this, finding that heightened levels of climate change awareness among university students can lead to increased sustainable behaviors. The studies by Song et al. (2022) and Li et al. (2022) build upon these ideas in their discussions by noting the role that higher education institutions can play in promoting and fostering sustainable behaviors among university students.

### Instagram in behavior change campaigns

A majority (71%) of 18- to 29-year-olds say they use Instagram (Pew Research Center, 2021), making the platform an apt choice to target college students. Given that this campaign was implemented during the COVID-19 pandemic, when students were learning remotely and opportunities to engage in person were not available, the use of a social media platform, such as Instagram, was even more important to increase engagement. The use of Instagram in communication campaigns designed to promote and sustain positive health behaviors is well-documented (Santarossa and Woodruff, 2018; Pilgrim and Bohnet-Joschko, 2019). Instagram has also been utilized as a communication tool for large public health authorities, like the Centers for Disease Control and Prevention (CDC; Kim and Kim, 2020).

Utilization of Instagram to promote positive behaviors among a target audience of university students has achieved notable success. Al-Eisa et al. (2016) found that Instagram was an effective tool for both motivating and maintaining healthy levels of physical activity among college students. While the above-mentioned studies are health behavior interventions that are not directly related to the topic of climate change, the findings from these studies are relevant to this campaign because they demonstrate the idea that Instagram can be used effectively as a tool to promote, motivate, and maintain positive behavioral changes among university students.

### Theory of planned behavior constructs

We borrowed constructs from the Theory of Planned Behavior (TPB; Ajzen, 1991) to develop this campaign. The TPB argues that in order to alter the behavior of a target audience, a campaign must be able to affect a change in the behavioral intentions of that target audience (Ajzen, 1991; Ajzen, 2020). In other words, behavioral intention is positioned as the most proximate, direct predictor of behavior (Ajzen, 2020). Behavioral intention refers to “the motivational factors that influence a behavior; they are indications of how hard people are willing to try, of how much of an effort they are planning to exert, in order to perform the behavior” (Ajzen, 1991, p. 188–189). The other constructs within the theory (attitudes, subjective norms, and perceived behavior control) are assumed to influence behavioral intent that, in turn, influences behavior, with intention as the immediate antecedent of behavior (Ajzen, 1991; Ajzen, 2020). Attitudes refer to “the degree to which a person has a favorable or unfavorable evaluation or appraisal of the behavior in question,” subjective norms are “the perceived social pressure to perform or not to perform the behavior,” and perceived behavioral control is defined as “the perceived ease or difficulty of performing the behavior” (Ajzen, 1991, p. 188). In addition to predicting behaviors, the TPB has proven to be successful in informing the design of behavior change interventions targeting groups (Steinmetz et al., 2016). Our campaign, inspired by these TPB constructs, aimed to influence the subjective norms and perceived behavior control of our target audience to affect a change in behavioral intent and a subsequent change in the target behavior.

#### Pro-environmental behaviors and the TPB constructs

Constructs from the TPB have been used extensively to measure and to predict pro-environmental behaviors (Heath and Gifford, 2002; Bamberg et al., 2003; Tikir and Lehmann, 2011; Greaves et al., 2013; Lin, 2013; de Leeuw et al., 2015; Elias et al., 2019; Pop et al., 2020; Aziz et al., 2021). In their study focusing on social media and sustainable purchasing behaviors, Pop et al. (2020) demonstrated the predictive ability of constructs from the TPB on intention to purchase green products. Meanwhile, Tikir and Lehmann (2011) demonstrated the effect of several TPB constructs on intentions to perform climate-friendly transportation behaviors. Further, the study by Aziz et al. (2021) builds upon these ideas, reitoriating the ability of several TPB constructs to predict intentions toward sustainable behaviors among employees in a university context.

#### University students and the TPB constructs

Constructs from the TPB have also been widely used in interventions within populations of university students (Namkoong et al., 2017; Record et al., 2017; Norman et al., 2018). Parrott et al. (2008) highlighted the potential for using TPB constructs to guide web-based interventions targeting college students. The TPB constructs have also been used to great effect in campaigns aimed at reducing binge drinking (Norman et al., 2018) and reducing smoking behaviors (Namkoong et al., 2017; Record et al., 2017) among university students. In particular, Namkoong et al. (2017) highlighted the role that subjective norms played in influencing behavioral change during their anti-smoking intervention. Accordingly, we did not necessarily design this study to test the TPB, but rather used TPB constructs as a guide to design the campaign materials, with the goal of the study being to evaluate the effectiveness of the campaign in changing behavior and in shifting cognitions related to the TPB constructs.

### Rationale and hypotheses

With growing concerns about the impacts of climate change, efforts to change individual behaviors at a larger scale need to be undertaken. We aimed to alter the constructs of subjective norms and perceived behavioral control in our target population to affect a change in behavioral intentions, and a subsequent change in behavior. While we recognize that past scholarship has divided normative beliefs into various subcategories - e.g. injunctive beliefs and descriptive beliefs - (Ajzen, 2020), in the present study, we decided to conceptualize and assess subjective norms as one holistic construct. We did so following the precedent set by previous scholarship which studied the TPB constructs in relation to sustainable behaviors (Bamberg et al., 2003; Greaves et al., 2013; Pop et al., 2020). Accordingly, we proposed the following hypotheses to evaluate the effectiveness of our TPB-inspired campaign.

*H1*: Compared to those who were not exposed to the campaign, those exposed to the campaign will show greater:

- Perceptions of subjective norms about engaging in sustainable behaviors.
- Perceived behavioral control over engaging in sustainable behaviors.
- Perceived behavioral intent to engage in sustainable behaviors in the future.
- Actual knowledge about sustainable behaviors.
- Perceived knowledge about sustainable behaviors.

We also anticipated that the number of sustainable behaviors self-reported via a campus website would be greater during the term while the campaign was running compared to the previous term, which was how our campus partner gaged sustainable behaviors on campus.

*H2*: The number of sustainable behaviors self-registered by campus community members will be greater during the academic term of the campaign compared to the previous academic term.

## Materials and methods

### Campaign description

This study was part of a student-based project at a small liberal arts university (~5,500 undergraduates, ~3,000 graduates) in Northern California in which senior class communication students were paired with a campus organization to develop and evaluate a campaign based on a behavior change theory and the goals and objectives of the campus partner. We collaborated with The Center for Sustainability, an organization on campus specifically focused on promoting sustainability at the University and in the community. The campus partner leads a number of initiatives aimed at promoting sustainability. Inside the classroom, the organization works with professors in order to integrate sustainability into the curriculum of classes across the university. Outside of the classroom, the organization hosts programs ranging from a student ambassador training program to an on-campus organic garden. The campus partner had also obtained a notable presence on social media with 2,171 followers on Instagram, 2,232 followers on Twitter, and a newsletter that reaches over 6,700 recipients monthly.

The campus partner has a branded program called the Sustainability Playbooks where they recommend 50+ sustainability behaviors across nine domains. Each behavior is correlated with one of the SDGs, representing a local contextualization of the overarching climate change mitigation goals that is specifically suited to the campus and student population. While some of these sustainable behaviors involve political and community action that may eventually affect institutional level change, the core of every recommended sustainable behavior lies in individual action. For example, the “transportation” domain includes a recommended behavior which encourages individuals to walk or ride their bike for their commute instead of driving a car. One of the key components of this program is a web portal wherein students and other campus community members can register their completion of the recommended sustainability behaviors.

Given the extensive work of The Center for Sustainability, internal data from the Office of Institutional Research between 2015 and 2019 has consistently shown that over 90% of students show positive attitudes toward sustainability, and over 60% of students believe that individuals can do something to make a difference.^1^ Accordingly, our campaign did not target attitudes about sustainability, and focused on motivating the student population to implement these recommended individual sustainable behaviors.

Communication materials designed for the campaign were mapped onto one of the various constructs from the TPB, including: 5 “Sustainability Icon” videos (subjective norms), 4 Instagram story posts (2 corresponding to PBC and 2 corresponding to subjective norms), and 4 Instagram feed posts (2 corresponding to PBC and 2 corresponding to subjective norms; see Table 1). For example, the video content reflected the concept of subjective norms by showcasing students completing sustainability behaviors, in order to present the behaviors as fun, popular activities that align with the culture of our campus community. The various subjective norms posts also focused on the fact that completing the behaviors will allow students to earn “sustainability badges,” messaging which was intended to highlight the badges as a form of status symbol connected to the environmentally sustainable norms of the campus community. On the other hand, the materials corresponding to PBC highlighted the sustainability behaviors as easy and quick actions: with phrases like “It’s so easy to earn an Energy Playbook badge!” Further, while the communication materials for the first week of the campaign promoted the sustainability behaviors program in general – without specifying a specific recommended behavior – the following 3 weeks promoted the purchasing, transportation, and energy domains, respectively.

**Table 1:** Communication materials.

TPB construct	Post type	Number of posts	Example
Subjective Norms	Sustainability Icon Video	5	Our first video aimed to promote plant-based food consumption. The video highlights key ingredients and provides a tutorial for preparing a plant-based recipe.
	Instagram Feed Post Instagram Story Post	2 2	We designed an image showcasing a woman riding a bike through a green space in a city. This digital illustration also included the transportation badge icon from the Sustainability Playbooks program to highlight the association of this behavior with the transportation domain of the Playbooks.
PBC	Instagram Feed Post Instagram Story Post	2 2	We designed a story post that showcased a woman standing near a refrigerator in front of a blue background. The post included the following text: “It’s so easy to earn an Energy Playbook badge!.” The post also included the badge icon for the energy domain of the Playbooks.
	Flyer	4	The flyer included a short message explaining the Sustainability Playbooks program as simple and easy actions which can be used to mitigate climate change.

Our communication materials were primarily distributed on Instagram by our campus partner, as well as 6 student-led organizations, who “reposted” our content, over the course of 4 weeks. These student organizations also promote pro-environmental missions, allowing them to leverage their existing networks to further the reach of our materials. Additionally, the campaign included one virtual flyer, targeting the construct of PBC, which was distributed via email listservs to student mailing lists.

### Participants/data

#### Exposure to campaign

The campaign was carried out on the public Instagram accounts of our campus partner and of our 6 student-led organizations, meaning that anyone could visit the pages and be potentially exposed to the campaign materials. At the time of the campaign, the combined reach of the participating Instagram accounts was ~4,250 followers. Flyers were also distributed via various student email listservs, many of which registered students are automatically enrolled.

#### Evaluation study

Through collaboration with the Office of Assessment at the University, our campaign evaluation measures were included on a regularly scheduled student-body-wide campus wellness assessment. The Institutional Review Board of the University approved the study before data collection began. An email invitation to complete the survey was sent out to all registered undergraduate students (*N* = 5,505) describing the purpose of the study and providing instructions on how to complete the survey. Those students who chose to participate were directed to a consent form. Once consent was given, participants completed 60 items on the survey. While the 60 total survey items on the assessment covered a range of topics relating to wellness and life on campus, we utilized only the demographics data and the data from the specific measures relating to the present study in our analysis (see Table 2). 1,552 participants completed the survey for a response rate of 28%. Records were also gathered from our campus partner on registered sustainability behaviors. The data that support the findings of this study are openly available in figshare [https://doi.org/10.6084/m9.figshare.16583600.v1].

**Table 2:** Measures.

Variables	Survey measures
Campaign Exposure	*“Have you seen any information on campus-based social media accounts, newsletters, or other sources about The Center for Sustainability’s sustainability behaviors?*” Yes / No / Not Sure
Subjective Norms	*1. Most people who are important to me and whose opinions I value would support me completing recommended sustainability behaviors.* *2. Most people who are important to me and whose opinions I value think that I should help to mitigate climate change by completing recommended sustainability behaviors* *3. Most people who are important to me and whose opinions I value would encourage me to help mitigate climate change by completing recommended sustainability behaviors.* Strongly Agree (1) / Strongly Disagree (7); Cronbach’s alpha = 0.88
Perceived Behavioral Control	*1. Whether or not I complete a recommended sustainability behavior is completely up to me.* *2. I am confident that if I want, I can complete a recommended sustainability behavior.* *3. I have resources, time, and opportunities to complete a recommended sustainability behavior.* Strongly Agree (1) / Strongly Disagree (7); Cronbach’s alpha = 0.69
Behavioral Intent	*1. I am willing to complete a recommended sustainability behavior and earn a Badge from the Sustainability Center.* *2. I plan to complete a recommended sustainability behavior and earn a Badge from the Sustainability Center.* *3. I will make an effort to earn Badges from the Sustainability Center during my time at [redacted]* Strongly Agree (1) / Strongly Disagree (7); Cronbach’s alpha = 0.89
Actual Knowledge	*“What are the Sustainability Playbooks?”* 1 = “A set of actions recommended by [redacted] Center for Sustainability that students can take to directly contribute to a more sustainable campus and world.” 2 = “A course at [redacted]” 3 = “A registered student organization at [redacted]” 4 = “Do not know.” *This item was recoded to indicate a correct answer to the question, such that the correct answer (option 1) was recoded as a “1” and the incorrect answers were coded to “0.”*
Perceived Knowledge	“*Please choose the one best response: Over Winter Quarter, my knowledge about how I can engage in sustainability behaviors has…,”* 1 = “Stayed the same as before” 2 = “Increased slightly” 3 = “Increased somewhat” 4 = “Increased significantly”

### Measures

#### Independent variable

##### Campaign exposure

Campaign exposure was assessed using a self-report item (see Table 2) with no, yes, and not sure as the possible response options. We decided to use three exposure categories because the “not sure” group is qualitatively different from a definitive “no” or a definitive “yes” in terms of recognition of exposure to the campaign.

#### Dependent variables

##### Subjective norms

Participants responded to a set of statements utilizing a 7-point, interval level, Likert-type scale (see Table 2). The first two measures were adapted from Bamberg et al. (2003) and the third measure was adapted from Han et al. (2010). The three items were combined to create a subjective norms scale (Cronbach’s alpha = 0.88).

##### Perceived behavioral control

For perceived behavioral control, participants responded to the three statements (Table 2). These measures for perceived behavioral control were adapted from Han et al. (2010)‘s study. These items were combined to create a scale (Cronbach’s alpha =0.69).

##### Behavioral intent

The measures designed to assess behavioral intent were also assessed on a 7-point, Likert-type scale (Table 2). These measures were also adapted from Han et al. (2010)‘s study. These items were combined to create a scale for behavioral intent (Cronbach’s alpha = 0.89).

##### Actual knowledge

The actual knowledge measure for our survey (see Table 2) was recoded to indicate a correct answer to the question, such that the correct answer (option 1) was recoded as a “1” and the incorrect answers were coded to “0.”

##### Perceived knowledge

We also included a self-reported knowledge item in our survey materials (Table 2). Assessing awareness with both a self-reported and actual knowledge assessment helped to give us a more holistic picture of the effect that our campaign may have had on increasing awareness of the Sustainability Playbooks.

##### Behaviors

The data from our campus partner tracked the details of every sustainability behavior registered on their website. The data includes the following variables: Role (e.g., student, alumni, faculty, etc.), Student Year (if applicable), and Date Completed. While the survey data were all collected from students on campus, the data on sustainability behaviors from our campus partner reflect the performance of sustainability behaviors registered by anyone in the campus community, including faculty, staff, alumni, and others.

##### Sociodemographics

In terms of demographic data, measures were included for the following categories: gender identity (*male, female, transgender female, transgender male, gender non-conforming, other, prefer not to answer*), race/ethnicity (*African-American/Black, Asian, Native Hawaiian/Pacific Islander, Caucasian/White, Hispanic/Latino, Spanish, Middle Eastern, Mixed Race, Other*), Housing Situation (*On-Campus Housing, Off-Campus Housing, In family home within US, In family home outside of US, other*), age (*years*), and class year (*first year, sophomore, junior, senior*).

### Data analysis plan

Descriptive statistics were computed for sample demographics. One-way ANOVAs were computed to assess differences between the three exposure groups on our key outcomes of subjective norms, perceived behavioral control, behavioral intent, and perceived knowledge. Post-hoc tests (Sheffe) were then conducted to further assess differences between pairs of groups. For the actual knowledge item, we utilized the re-coded actual knowledge item to run a Chi-Square test in SPSS. All tests were conducted using SPSS 27, with a significance level set at *p* < 0.05. The data that support the findings of this study are available here: https://doi.org/10.6084/m9.figshare.16583600.v1.

## Results

### Descriptive results

Data were collected from 1,552 participants (28% response rate). Of these respondents, 29.1% were first-year students, 24.1% were Sophomores, 25.8% were Juniors, and 21.0% were Seniors (See Table 3). In terms of gender and race/ethnicity, the sample was 56.4% female and Caucasian/White (50.1%). With respect to geography, most students were living either off campus on their own (36.7%) or with their family (53.2%). The mean age of the sample population is 19.9 years old.

**Table 3:** Descriptive statistics (*n* = 1,552).

Gender	
Male	42.1%
Female	56.4%
Transgender Female	0.1%
Transgender Male	0.1%
Gender Non-conforming	0.7%
Other	0.2%
Preferred Not to Answer	0.4%
Race/Ethnicity	
African American / Black	3.2%
Asian	24.9%
Native Hawaiian / Pacific Islander	0.6%
Caucasian / White	50.1%
Hispanic/Latino/Spanish	10.8%
Middle Eastern	1.3%
Mixed Race	7.4%
Other	1.7%
Academic Year	
First-years	29.1%
Sophomores	24.1%
Juniors	25.8%
Seniors	21.0%
Housing Situation	
On-Campus Housing	4.4%
Off-Campus Housing	36.7%
In my family home or residence within the US	53.2%
In my family home or residence outside of the US	5.1%
Other	0.6%
Age	Mean = 19.95
	SD = 1.89
	Range = 17–51
Exposure to Campaign	
Yes	44.7%
No	31.0%
Not Sure	24.3%

### Exposure to campaign

When assessing exposure to the campaign materials, the participants were divided into three groups: Yes (Exposure Group), No (Non-exposure Group), and Unsure. The Exposure Group was the largest with 44.7% of respondents, followed by the Non-exposure Group at 31.0%, and the Unsure Group at 24.3%.

### Subjective norms

The results from a one-way ANOVA showed that the Exposure Group reported the highest scores for subjective norms with a mean score of 5.93 (SD = 0.99; see Table 4). The Unsure Group reported the second-highest scores for subjective norms, with a mean score of 5.66 (SD = 1.08). The Non-exposure Group reported the lowest scores for subjective norms, with a mean score of 5.54 (SD = 1.23). The difference in mean score between the Exposure Group and the Non-exposure Group was 0.39 [*F*(2,717) = 7.86, *p* < 0.001].

**Table 4:** One-way ANOVA.

	Yes	Unsure	No	df	*F*	*n*	*p*
Subjective norms	5.93	5.66	5.54	2	7.86	720	**0.000**
PBC	5.71	5.47	5.46	2	2.59	519	0.076
Behavioral intent	4.58	3.97	3.58	2	24.8	633	**0.000**
Self-reported knowledge	1.72	1.43	1.39	2	20.9	1,109	**0.000**

The bold values represent the statistically significant of *p* values.

Post-hoc analyses using the Sheffe showed that subjective norms were greater in the Exposure Group compared to the Non-exposure Group (*p* < 0.001). Differences between the “Not Sure” vs. the Exposure Group and the “Not Sure” vs. the Non-exposure Group were not significant (*p* = 0.06 and *p* = 0.55, respectively). The Sheffe test was selected due to the unequal sample sizes, and because we did not have planned contrasts. Thus, H1a was supported.

### Perceived behavioral control (PBC)

The Exposure Group reported the highest scores for PBC with a mean score of 5.71 (SD = 0.93), followed by the Unsure Group (5.47, SD = 103). The Non-exposure Group reported the lowest scores for PBC, with a mean score of 5.46 (SD = 1.16; see Table 4). The difference in mean score between the Exposure Group and the Non-exposure Group was 0.25 [*F*(2, 516) = 2.59, *p* = 0.076]. H1b was not supported.

### Behavioral intent

The one-way ANOVA [*F*(2, 717) = 24.88, *p* < 0.001] showed that the Exposure Group reported the highest mean scores for behavioral intent (4.58, SD = 1.56; see Table 3). The Unsure Group reported the second-highest scores for behavioral intent (3.97, SD = 1.36), and the Non-exposure Group had the lowest scores (3.58, SD = 1.53). The difference in mean score between the Exposure Group and the Non-exposure Group was 1.00, which was the largest of any of the TPB constructs that we assessed. Post-hoc analyses using the Sheffe showed that behavioral intentions were greater in the Exposure Group compared to the Non-exposure Group (*p* < 0.001) as well as the Unsure Group (*p* < 0.001). Differences between the “Not Sure” vs. the Non-exposure Group was also significant (*p* = 0.03). Based on these results, H1c was also supported.

### Actual knowledge

The Chi-Square analysis showed that the Exposure Group had the highest percentage of correct actual knowledge scores, at 48.9% [*Χ^2^* (2, *N* = 1,110) = 154.98, *p* < 0.0001; Table 5]. The Non-exposure Group reported the second-highest percentage of correct actual knowledge scores, at 29.3%. The Unsure Group scored the lowest percentage of correct actual knowledge scores, at 20.9%. Across all three groups, 44.8% of the sample population identified the correct answer. The difference in percentage correct score between the Exposure Group and the Non-exposure Group was 19.6%. Post-hoc analyses using the Sheffe showed that knowledge was greater in the Exposure Group compared to the Non-exposure Group (*p* < 0.001) as well as the Unsure Group (*p* < 0.001). Differences between the “Not Sure” vs. the Non-exposure Group was also significant (*p* = 0.04). Thus, H1d was supported.

**Table 5:** Actual knowledge chi square test.

	Total	Correct % (*n*)	Incorrect % (*n*)
Exposure (Yes)	344	49.8% (248)	15.7% (96)
Non-Exposure (No)	495	29.3% (146)	57.0% (248)
Not Sure	271	20.9% (104)	27.3% (167)

### Self-reported knowledge

The results of the ANOVA [*F*(2, 1,106) = 20.97, *p* < 0.0001] for self-reported knowledge data show that the Exposure Group reported the highest levels of self-reported knowledge with a mean score of 1.72 out of 4 (SD = 0.87; Table 4). The Unsure Group reported the second-highest levels of self-reported knowledge, with a mean score of 1.43 (SD = 0.68). The Non-exposure Group reported the lowest levels of self-reported knowledge, with a mean score of 1.39 (SD = 0.70). The difference in mean score between the Exposure Group and the Non-exposure Group was 0.33. H1e was supported.

### Behavior

We hypothesized that the number of self-reported sustainable behaviors on the registration database would be greater during the academic term of our campaign compared to the previous academic term. 48 behaviors were registered during the term featuring our campaign (Winter 2021), compared to 8 behaviors in the Fall 2020 term (Table 6). Hypothesis 2 was supported by our results.

**Table 6:** Number of sustainable actions registered during COVID 19 remote learning.

Academic Term	Dates	# of Registered Behaviors
Spring 2020	30 Mar 2020–11 June 2020	14
Fall 2020	21 Sept 2020–11 Dec 2020	8
Winter 2021	4 Jan 2021–19 Mar 2021	48*
Spring 2021	29 Mar 2021–10 June 2021	9
Fall 2021	20 Sept 2021–10 Dec 2021	11

* indicates the academic quarter during which the communication campaign was conducted.

Compared with other quarters of remote learning, the two quarters preceding our campaign had 14 behaviors (Spring 2020) and 8 behaviors (Fall 2020), respectively; the two subsequent quarters had 9 behaviors (Spring 2021) and 11 behaviors (Fall 2021), respectively. The academic quarter which featured our campaign (Winter 2021), saw 48 registered behaviors: higher than two previous and two subsequent quarters of remote learning (see Table 6).

## Discussion

The purpose of this study was to assess the effectiveness of an Instagram campaign which utilized constructs from the Theory of Planned Behavior to promote sustainability behaviors among undergraduates. We found that of the 1,552 respondents on the survey, 44.7% reported seeing our campaign, 31% reported not seeing our campaign, and 24.3% were not sure. As has been suggested by Segerberg (2017), putting information about climate change online does not guarantee attention among the target audience; thus we believe a 44.7% self-reported exposure rate is reasonable.

We found that individuals who had been exposed to our campaign reported higher mean scores for subjective norms and behavioral intent compared to those who were not exposed to our campaign. Behavioral intent had the largest difference in mean score between the Exposure Group and Non-exposure Group. Those who were exposed to our campaign also showed greater knowledge about the target sustainability behaviors than those who were not exposed. While the differences between groups for subjective norms and knowledge were small, previous meta-analyses on media campaigns for health have shown similar effect sizes (see Noar, 2007). These findings provide evidence for the effect that our campaign may have had in increasing knowledge of, and intention to participate in, sustainable behaviors.

Conversely, the results for PBC had the smallest difference in mean score (0.25) and were not statistically significant (*p* = 0.076). Previous studies that have utilized TPB constructs have also seen smaller effects for PBC than other constructs (Namkoong et al., 2017; Norman et al., 2018). For example, the study by Namkoong et al. (2017) found that their intervention did not successfully affect the construct of PBC (*p* = 0.94), despite having a significant impact on subjective norms (*p* = 0.05). We suspect that we saw a smaller effect for PBC in our study partially due to the fact that we implemented the campaign during the COVID-19 pandemic. Looming financial concerns, lack of complete information about the effects of the virus, in addition to the isolation many faced, may have led to an overall generalized sense of lack of control (Wnuk et al., 2020), and may have influenced the sense of control over sustainable behaviors among our participants.

We also included observational data as a part of our study. While data from the sustainability behaviors registry did not directly match the number of participants who responded to the survey with intentions to engage in sustainable behaviors, the rate of increase in self-registrations of behavior during the time of the campaign was substantially greater than the previous term (48 in Winter 2021 compared to 8 in Fall 2020). The purchasing domain, which we targeted first, showed the highest number of actual registered behaviors during the campaign period: constituting 12 of the 48 total registered behaviors. Further, 11 of those 12 purchasing behaviors were registered for the specific action that we promoted in our communication materials (consuming vegetarian or vegan meals). Although we also targeted the energy and transportation domains, the outcomes for these areas were less pronounced, with 2 and 1 registered behaviors, respectively.

Overall, these are much lower than the numbers from the previous academic year, however, we suspect that students learning remotely due to the COVID-19 pandemic may have tempered the enthusiasm and engagement for the program. Internal data from our Office of Assessment showed that about 30% of students had not participated in any campus activities after the end of the Fall 2020 quarter, during which we were fully remote. Reasons for this lack of participation included Zoom fatigue, overwhelm from coursework, and difficulty finding activities related to interests. Thus, the increase in the number of self-registered behaviors we saw from the previous term may be an even stronger indication of the campaign’s success. Additionally, in a typical year, our campus partner held multiple on-campus events that could have contributed to increased participation in behaviors that qualified for registration. Early research on the effects of the COVID-19 pandemic at higher education institutions has shown decreased connection between students and their on-campus community, and changes to extracurricular participation (Gonzalez-Ramirez et al., 2021; Lederer et al., 2021).

It is possible that participants engaged in sustainable behaviors, but did not register them. Each of the results we found for the TPB constructs lend their support to this idea and the findings for behavioral intent are particularly valuable in supporting this notion, as behavioral intent is the strongest predictor of actual behavior (Ajzen, 1991; Ajzen, 2020). While we did not assess self-reported behaviors in the survey, correlational studies have consistently shown that intentions are reliably associated with behavior. In a meta-analysis of 185 studies that have used the TPB, the average correlation between measures of intention and behavior was .47 (Armitage and Conner, 2001). Additionally, a meta-analysis of experimental studies found that changes in intention lead to changes in behavior, though the size of this effect is considerably smaller than correlational tests have suggested (Webb and Sheeran, 2006). This may explain why even though we saw a high score on behavioral intention in our self-reported survey data, this did not necessarily translate into the observed behavioral registry data.

What we have offered here is not necessarily a test of the predictive power of the antecedent constructs of subjective norms and perceived behavioral control on behavioral intent, but rather, an evaluation of whether a communication campaign developed using constructs borrowed from the TPB, and delivered via Instagram, was effective in shaping the cognitive responses related to subjective norms, self-reported knowledge, and perceived behavioral intention to engage in sustainable behaviors of those who saw the campaign. Our results suggest that an Instagram campaign can work to move peoples’ cognitions about engaging in sustainable behaviors in the desired direction. This echoes the success of decades of work in the use of media campaigns to change health behaviors (see Wakefield et al., 2010) and suggests that employing strategies from behavior change campaigns may be one avenue toward encouraging more behavior change with regard to sustainable behaviors to mitigate climate change.

Our campaign was a fully virtual campaign, delivered via Instagram and email listservs, which focused on a specific target audience to engage in sustainable behaviors to mitigate climate change. A recent review of online and social media campaigns for climate change (Segerberg, 2017) highlighted that, “the study of online and social media campaigns to engage the public with climate change is still in its infancy” (p. 3). We are pleased to contribute work on what we believe to be one of the first studies to present an evaluation of a successful campaign to shift cognitions related to sustainable behaviors delivered via Instagram. This complements the wide range of work that has been done to raise awareness and shape scientific understanding among publics and mobilize citizens in pressuring decision makers to implement climate change mitigation policies (see Robelia et al., 2011; Senbel et al., 2014; Segerberg, 2017).

Our campaign targeted college students, because previous research has shown that they may hold favorable attitudes regarding sustainable behaviors (Carducci et al., 2021) and heightened levels of awareness about climate change (Jamelske et al., 2013; Wachholz et al., 2014; Aksit et al., 2017; Mazo and San Juan, 2019). Focusing on closely networked individuals, such as students on a college campus with a cohesive culture, as we have done in this campaign, aligns with work that has found linkages between networks, conversations within networks, and attitudes and beliefs about climate change. As Leombruni (2015) found, culturally grouped individuals talking about climate change can lead to others in the group to adopt the same opinions as projected by the group identity. College campuses present an ideal setting for sustainability and climate change campaigns; they culturally group individuals who already show an awareness of climate change (Mazo and San Juan, 2019), positive attitudes about sustainable behaviors (Carducci et al., 2021), and are networked together. We hope that our work can serve as a model for how to engage students on campuses across the world.

### Limitations and future directions

One major limitation of this study is that, with the non-experimental design we utilized in our campaign, we were unable to effectively control for exposure and utilized a self-reported measure. We were also unable to collect pre-test data and compare to post-test data, resulting in a cross-sectional design. However, we believe our study still presents valuable findings and proposes a novel way for scholars, students, and sustainability-focused organizations to partner and work together.

Another limitation relates to the use of digital dissemination channels - while the vast majority of the campaign’s communication materials were distributed via Instagram, there was one promotional flyer that was also distributed via email listservs to the student population. This distribution through various channels, along with resharing and reposting of Instagram materials made by the general student population, also represents a potential limitation for the study. With various user-focused algorithms at play, we could not control who viewed the campaign versus not, as well as who shared the materials versus not. However, the viewing and resharing of materials through various channels is a prevalent aspect of social networks and, while the lack of experimental control in this authentic online environment certainly constitutes a limitation, it adds to the study in other ways. Environmental communication, which aims to positively influence individual behaviors, is only valuable in that it can change the behaviors of everyday people in a real-world setting. The fact that this campaign achieved success in the “real world,” outside of a controlled experimental setting, suggests strong external validity for the strategies we utilized in other real-world environments.

We also did not measure attitudes or self-reported behavior. For attitudes, we knew from internal data from the university that attitudes toward sustainability were already high among students; however, measuring this construct may have allowed us to conduct more rigorous analyses in testing the TPB as a model. For behaviors, we attempted to triangulate data from our campus partner to account for behavior; however, because the behaviors were self-reported via the registration database, we were unable to fully triangulate the results. Relatedly, with the use of the registration data and our non-experimental design, we were unable to ensure that the same specific participant individuals had registered behaviors in each of the respective academic terms; nor were we able to track specific individuals’ changes in their number of registered behaviors in the database. In the future, we may measure self-reported behavior on our evaluation survey, as well as examine analytics data from the campus partner’s website and Instagram accounts. We did not include analytics from Instagram in our formal evaluation plan, but future research should certainly take advantage of the potential for analytics that come from the campaign distribution platform, as these analytics could serve as an additional data point for exposure (see Grantham et al., 2021). Despite these limitations, our study makes an important contribution in helping us understand how college-campus based campaigns can be effective in shifting the cognitive processes underlying behavior change related to climate change mitigation efforts.

### Practical implications

The findings from this study have a number of implications for behavior change interventions. First, our results highlight the utility of behavior change theories in the design of environmental communication interventions. While many studies have tested the TPB in relation to the topics of climate change and sustainable behaviors (Tikir and Lehmann, 2011; Lin, 2013; Aziz et al., 2021), very few have designed, executed, and evaluated actual climate change mitigation campaigns which drew from these theories. Our results suggest that TPB constructs can be utilized to inform successful communication interventions aimed at promoting sustainable behaviors. This has widespread implications for the use of behavioral change theories in environmental communication interventions; further investigation is warranted.

Our results have implications for the structure and execution of behavior change interventions and environmental communication practice in university contexts. Our study presents an evaluation of a communication campaign developed through a campus organization-student-faculty partnership. As the effects of climate change continue to worsen, universities and their community members are increasingly taking responsibility for assessing and reducing their own carbon footprints (Sippel et al., 2018; Clabeaux et al., 2020; Yusoff et al., 2021). The campaign evaluated in this study can serve as a model for colleges and universities aiming to reduce their carbon footprint and/or expand their on-campus sustainability efforts.

Beyond the student research team and faculty advisor, the distribution of our communication campaign involved one on-campus partner organization and six on-campus student-led organizations, all of which already had large followings on Instagram or via email listservs. Identifying student organizations with large followings on campus and partnering with these organizations could be beneficial to increasing the reach of behavior change campaigns. The participation of students and on-campus organizations in the distribution of pro-environmental communication campaigns may also play a role in establishing pro-environmental norms on a university campus: potentially bolstering attempts to affect the construct of subjective norms with a communication intervention. Future research should explore additional avenues of engaging students and on-campus organizations in the design and implementation of pro-environmental behavior change communication interventions on college and university campuses.

## Conclusion

As the climate crisis continues to worsen, more strategies to mitigate climate change are needed, including encouraging individuals to engage in actions that will lead to more sustainable lifestyles. Our campaign, developed with constructs borrowed from the Theory of Planned Behavior, and implemented via Instagram at a small liberal arts university, showed success in shifting sustainability-related cognitions and behaviors. Compared with the Non-exposure Group, participants with self-reported exposure to the campaign materials held higher subjective norms about and higher intent to perform sustainable behaviors, as well as greater knowledge of the sustainable behaviors promoted by the campaign. Additionally, observational data from an on-campus registry showed an increase in registered sustainable behaviors during the course of our campaign, when compared to the previous and subsequent academic terms of remote learning. We believe that our campaign can serve as a model for other universities in their efforts to promote individual sustainable behaviors in their local communities. With the motivation of sustainable behaviors en masse being a crucial aspect of mitigating climate change, our campaign provides a guide for future interventions performed at greater scales, among universities and the general public, which will forge a vital component of the global effort to combat the climate crisis.

## Data availability statement

The datasets presented in this study can be found in online repositories. The names of the repository/repositories and accession number(s) can be found below: https://doi.org/10.6084/m9.figshare.16583600.v1.

## Ethics statement

The studies involving humans were approved by Santa Clara University Institutional Review Board. The participants provided their electronic informed consent to participate in this study.

## Author contributions

AV: Conceptualization, Investigation, Writing – original draft, Writing – review & editing. CT: Supervision, Writing – original draft, Writing – review & editing. SJ: Data curation, Project administration, Writing – review & editing.

## Footnotes

^1^Data from 2015, 2017, and 2019 show that over 90% of students agree that they have a personal responsibility to make a difference on sustainability related issues, and over 60% disagreed that there was little individuals can do to make a difference in sustainability.
